# Analysis of hot trends in research on the association between vitamin D and cardiovascular disease

**DOI:** 10.3389/fnut.2022.1073698

**Published:** 2023-01-13

**Authors:** Xuemei Luo, Feifeng Wu, Cheng Wang, Chuan Wen

**Affiliations:** Department of Pediatrics, The Second Xiangya Hospital, Central South University, Changsha, China

**Keywords:** vitamin D, cardiovascular disease, visualization studies, bibliometric analysis, bibliographic items co-occurrence matrix builder

## Abstract

**Objective:**

Vitamin D deficiency is the most common nutrient deficiency. Numerous studies suggest that vitamin D is an independent risk factor for cardiovascular disease. The objective is to visualize the research hotspots and evolution trends of the correlation between vitamin D and cardiovascular disease by using multivariate statistics and social network analysis techniques and to compare adult research with that of children in this field.

**Methods:**

(Vitamin D [MeSH Major Topic]) AND (cardiovascular disease [MeSH Major Topic]) were retrieved from the PubMed database by time period. The bibliographic items co-occurrence matrix builder (BICOMB) was adopted to extract high-frequency subject terms and establish the core subject term co-occurrence matrix. With the Netdraw function of Ucinet 6.0 software, the social network of core subject terms was completed.

**Results:**

Before 2010, there was a slow increase in the number of research papers covering all age groups in this field (157, 54, 84, and 211 papers were published in stages 1–4, respectively). From 2010 to 2020, there were 1,423 papers retrieved, showing a significantly increased research heat. The overall development trend of the research on the association between vitamin D and cardiovascular disease in children is similar to that in all age groups. From 2010 to 2020, 122 related papers were published (while before 2009, there were only 43 papers in all), presenting a good overall development trend. The social network analysis of core subject terms showed gradually increased correlations between research hotspots, from the early studies limited on the physiological function of vitamin D in cardiovascular diseases, to the role of vitamin D in the comorbidities of various cardiovascular diseases and its value as an intervention measure. Researches on the association between vitamin D and cardiovascular disease has a good overall development trend. Study of the mechanisms and the role of vitamin D in the common co-morbidities of cardiovascular disease and its therapeutic value will be the focus of future research.

## 1. Introduction

Vitamin D is a lipid steroid hormone with complex physiological functions. Of all nutritional deficiencies, vitamin D deficiency is the most common. Vitamin D deficiency has become one of the global public health problems. It is estimated that approximately 30% of children and 60% of adults worldwide suffer from vitamin D deficiency and insufficiency respectively (1). Vitamin D is considered an independent risk factor for cardiovascular disease (2–4). Vitamin D deficiency can lead to the occurrence of vascular calcification, calcium and phosphorus metabolism disorders (it may contribute to the progression of vascular calcification), impaired hemodynamic variables (further resulting in peripheral arterial diseases such as endothelial abnormalities, hypertension, ischemic cardiomyopathy, cardiomyopathy, myocardial hypertrophy, myocardial infarction, and atherosclerosis), insulin-resistant metabolic syndrome, and a further increase in the risk of hypertension and cardiovascular disease-related mortality (5). In addition to as a means of preventing and treating rickets, vitamin D has been tried in a variety of disease interventions, including cardiovascular disease (6). Researchers can read reviews and meta-literatures to understand the current state of research in this field, including the relevance of vitamin D to cardiovascular disease, mechanistic theories, and the predictive and interventional value of vitamin D in the onset and regression of cardiovascular disease (2, 5, 6). This paper attempts to provide a realistic, visual and longitudinal picture of the distribution and evolution of research hotspots in this field using multivariate statistics and social network analysis techniques (an important method in bibliometrics) to inform researchers in establishing research directions.

## 2. Materials and methods

### 2.1. Literature database search

All publications retrieved in the PubMed database using relevant subject terms were analyzed.

### 2.2. Principles for establishing core subject terms

The main subject terms with a word frequency greater than 0.5% were used as core subject terms for analysis.

### 2.3. Search strategy

#### 2.3.1. Subject terms

(Vitamin D [MeSH Major Topic]) AND (cardiovascular disease [MeSH Major Topic])

#### 2.3.2. Staged retrieval

The retrieval periods were classified according to natural chronology. The first stage was before 31 December 1979 (given the limited number of documents included in the PubMed database in the early years, the period before 1979/12/31 is classified as the same stage); the retrieval period for the second stage was (“1980/01/01”[Date-Publication]]: “1989/12/31”[Date-Publication]); the retrieval period for the third stage was (“1990/01/01”[Date-Publication]: “1999/12/31”[Date-Publication]); the retrieval period for the fourth stage was (“2000/01/01”[Date-Publication]: “2009/12/31”[Date-Publication]); and the retrieval period for the fifth stage was (“2010/01/01”[Date-Publication]: “2020/12/31”[Date-Publication]).

#### 2.3.3. Study objects

##### 2.3.3.1. Association between vitamin D and cardiovascular disease

No age filter was set.

##### 2.3.3.2. Study on the association between vitamin D and cardiovascular disease in children

**FIGURE 1:**
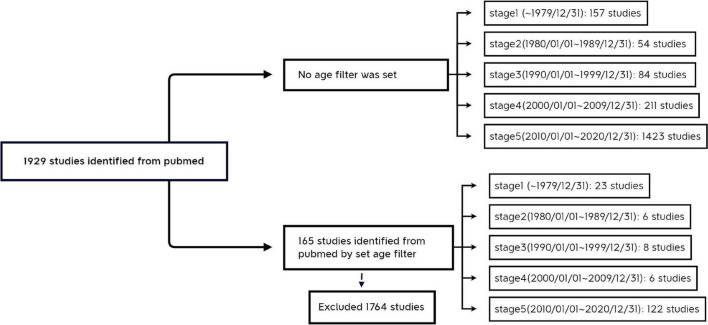
Research Roadmap.

“Child: birth–18 years” [MeSH Terms] OR “Newborn: birth–1 month” [MeSH Terms] OR “Infant: birth–23 months” [MeSH Terms] OR “Infant: 1–23 months” [MeSH Terms] OR “Preschool Child: 2–5 years” [MeSH Terms] OR “Child: 6–12 years” [MeSH Terms] OR “Adolescent: 13–18 years” [MeSH Terms] (Figure 1).

### 2.4. Multivariate statistical and social network analysis methods

The PubMed database search hits were imported into the Bibliographic Items Co-occurrence Matrix Builder (BICOMB) (7), and high-frequency subject terms were extracted from all subject terms at each stage of development and core subject terms were established to build a core subject term co-occurrence matrix. A social network of the co-occurrence matrix was drawn using the Netdraw function of Ucinet 6.0 software. In the social network diagram, the closer the nodes are to the center, the more central the term is in the whole social network; the larger the nodes are, the higher the frequency of the words; the thicker the line between the core words, the higher the degree of association between the two core words.

## 3. Results

### 3.1. General information of the retrieved literature

#### 3.1.1. Vitamin D AND cardiovascular disease

**FIGURE 2:**
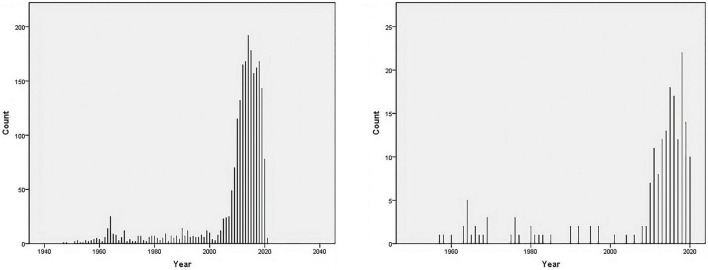
Number of publications on “vitamin D AND cardiovascular disease” in the PubMed database as of December 2020. **(A)** Vitamin D [MeSH Major Topic] AND cardiovascular disease [MeSH Major Topic]. **(B)** Vitamin D [MeSH Major Topic] AND cardiovascular disease [MeSH Major Topic], “Child: birth-18 years” [MeSH Terms] OR “Newborn: birth-1 month” [MeSH Terms] OR “Infant: birth-23 months” [MeSH Terms] OR “Infant: 1–23 months” [MeSH Terms] OR “Preschool Child: 2–5 years” [MeSH Terms] OR “Child: 6–12 years” [MeSH Terms] OR “Adolescent: 13–18 years” [MeSH Terms].

We searched the PubMed database and selected subject terms with a word frequency greater than 0.5% as the core subject terms. For the time period before 1979, 157 publications were retrieved, with 336 main subject terms; the lowest core subject term frequency was 2, and 41 subject terms were analyzed. A total of 54 publications from 1980 to 1989 were retrieved, with 527 main subject terms; the lowest core subject term frequency was 2, and there were 25 subject terms included in the analysis. From 1990 to 1999, 84 publications were retrieved, with 343 main subject terms; the lowest core subject term frequency was 2, and 46 subject terms were included in the analysis. For the time period from 2000 to 2009, 211 publications were retrieved, with 879 main subject terms; the lowest core subject term frequency was 5, and 34 subject terms were included in the analysis. For the time period from 2010 to 2020, 1,423 publications were found, with 5,822 main subject terms; the lowest core subject term frequency was 30, and 27 subject terms were included in the analysis (Figure 2 and Table 1).

**TABLE 1:** Literature analysis of “vitamin D AND cardiovascular disease” in PubMed as of December 2020.

Stage	No. of retrieved publications	No. of main subject terms	Social network analysis of core subject terms		
			Core	Second layer	Outer layer
∼1979	157	336	Vitamin D	Dihydrotachysterol	Rheumatic Heart Disease
				Calcinosis	Vitamin A
				Aortic Disease	Coronary Disease
				Aortic Valve Stenosis	Myocardial Infarction
				Hypercalcemia	Arteriosclerosis
				Cardiovascular Diseases	Pregnancy Complications, Cardiovascular
					Growth
					Pressoreceptors
					Multiple Myelom
1980–1989	54	527	Vitamin D	Hypertension	Calcitriol
				Calcium	Cardiovascular Diseases
					Cardiomyopathies
					Coronary Disease
					Multiple Myeloma
					Rats, Inbred Strains
1990–1999	84	343	Calcitriol	Hypertension	Multiple Myeloma
				Calcium	Antineoplastic Agents
				Vitamin D	Tunica Intima
				Parathyroid Hormone	Calcium Channel Agonists
					Immunosuppressive Agents
2000–2009	211	879	Vitamin D	Cardiovascular Diseases	Heart Failure
				Vitamin D Deficiency	Diabetes Mellitus, Type 2
					Peripheral Vascular Diseases
					Hypertrophy, Left Ventricular
					Neoplasms
					Myocardial Infarction
					Stroke
					Calcium Channel Agonists
					Coronary Disease
					Receptors, Calcitriol
					Blood Pressure
2010–2020	1423	5822	Vitamins	Vitamin D Deficiency	Brain Ischemia
				Cardiovascular Diseases	Renal Insufficiency, Chronic
				Atherosclerosis	Metabolic Syndrome
				Receptors, Calcitriol	Neoplasms
					Blood Pressure

#### 3.1.2. Vitamin D AND cardiovascular disease (0–18 years)

We searched the PubMed database and selected subject terms with a word frequency greater than 0.5% as the core subject terms. For the time period before 1979, 23 publications were retrieved, with 41 main subject terms; the lowest core subject term frequency was 1, and 25 subject terms were analyzed. A total of 6 publications from 1980 to 1989 were retrieved, with 16 main subject terms; the lowest core subject term frequency was 1, and there were 12 subject terms included in the analysis. From 1990 to 1999, 8 publications were retrieved, with 27 main subject terms; the lowest core subject term frequency was 1, and 17 subject terms were included in the analysis. For the time period from 2000 to 2009, 6 publications were retrieved, with 27 main subject terms; the lowest core subject term frequency was 1, and 19 subject terms were included in the analysis. For the time period from 2010 to 2020, 122 publications were retrieved, with 489 main subject terms; the lowest core subject term frequency was 3, and 23 subject terms were included in the analysis (Figure 2 and Table 2).

**TABLE 2:** Literature analysis of “vitamin D AND cardiovascular disease (0–18 years)” in PubMed as of December 2020.

Stage	No. of retrieved publications	No. of main subject terms	Social network analysis of core subject terms		
			**Core**	**Second layer**	**Outer layer**
∼1979	23	41	*Ascorbic Acid^1^		
			*Arteriosclerosis^2^		
			*Aorta, Abdominal^2^		
			*Ergocalciferols^3^	*Tachycardia^3^	
				*Cardiomyopathies^3^	
				*Cardiovascular Diseases^3^	
			*Vitamin D^4^	*Calcium^4^	*Kidney Diseases^4^
				*Hypercalcemia^4^	*Hypercalcemia^4^
				*Aortic Diseases^4^	*Rickets^4^
1980∼1989	6	16	*Calcitriol^5^	*Immunologic Deficiency Syndromes^5^	
				*DiGeorge Syndrome^5^	
			*Vitamin D^6^	*Aortic Valve Stenosis^6^	*Ergocalciferols^6^
				*Tachycardia^6^	*Coronary Disease^6^
				*Arteriosclerosis^6^	
1990∼1999	8	27	*Calcitriol^7^	*Hypertension^7^	
				*Vitamin D Deficiency^7^	
				*Parathyroid Hormone^7^	
			*Vitamin D^8^	*Kidney^8^	*Osteocalcin^8^
				*Arteriosclerosis^8^	*Osteoblasts^8^
				*Calcinosis^8^	*Turner Syndrome^8^
			*Cholecalciferol^9^	*Aortic Aneurysm, Abdominal^9^	
2000∼2009	6	27	*Calcitriol^10^	*Calcium Channel Agonists^10^	*Hyperparathyroidism^10^
				*Hypophosphatemia, Familial^10^	*Cardiomyopathies^10^
				*Calcinosis^10^	*Phosphates^10^
			*Vitamin D^11^	*Vitamin D Deficiency^11^	*Vascular Diseases^11^
				*Cardiovascular Diseases^11^	*Sunlight^11^
					*Autoimmune Diseases^11^
					*Metabolic Syndrome^11^
2010∼2020	122	489	*Vitamin D^12^	*Vitamin D Deficiency^12^	*Calcifediol^12^
				*Cardiovascular Diseases^12^	*Pediatric Obesity ^12^
					*Metabolic Syndrome^12^
					*Diabetes Mellitus, Type 1^12^
					*Parathyroid Hormone^12^
					*Endothelium, Vascular^12^
					*Mucocutaneous Lymph Node Syndrome^12^

*Previous research hotspots are relatively separated, so the subgroups are grouped together. Numbers 1–12 represent the different clusters they belong to.

### 3.2. Social network analysis of the core subject terms

#### 3.2.1. Vitamin D AND cardiovascular disease

**FIGURE 3:**
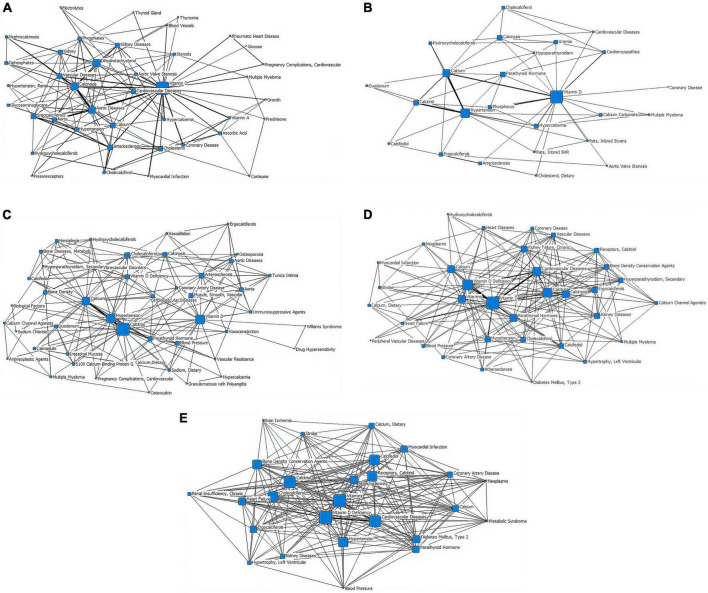
Social network of core subject terms of “vitamin D AND cardiovascular disease” in the PubMed database before December 2020. **(A)** 1979. **(B)** 1980-1989. **(C)** 1990-1999. **(D)** 2000-2009. **(E)** 2010-2020.

In the first stage (∼1979), “vitamin D” is at the core of the network, with a node area much larger than the other core subject nodes and closely linked to the other subject terms. The terms “dihydrotachysterol,” “calcinosis,” and “aortic diseases” have larger node areas in the second tier. There are relatively thicker lines between “calcinosis,” “aortic diseases,” and “vitamin D.” “Cardiovascular diseases,” “aortic valve stenosis,” and “hypercalcemia” are also in the second tier but have smaller node areas. In the outer layer, the terms “rheumatic heart disease,” “vitamin A,” “coronary disease,” “myocardial infarction,” and “arteriosclerosis” are closely linked to the core term “vitamin D,” while “pregnancy complications, cardiovascular,” “growth,” “pressoreceptors,” and “multiple myeloma” are weakly associated with “vitamin D” (Figure 3A and Table 1).

In the second stage (1980–1989), “vitamin D” remains at the heart of the network, with the largest node area and is strongly connected to other nodes around it. The core subject terms of the second tier are “hypertension” and “calcium,” both of which are closely related to “vitamin D.” The lines between “calcium,” “calcitriol,” and “hypertension” are all thick, forming sub-centers of the study. “Cardiomyopathies” and “cardiovascular diseases” are in the outer layer of the network, with a thicker line between “cardiomyopathies” and “vitamin D.” “Rats, Inbred Strains” appears in the outer network where “multiple myeloma” still exists (Figure 3B and Table 1).

In the third stage (1990–1999), “calcitriol” is the most central term with the largest node area, closely related to the secondary core subject terms of “calcium,” “hypertension,” and “parathyroid hormone.” The “vitamin D,” “hypertension,” and “calcium” nodes are large and closely related to the periphery and are presented as three sub-study centers. “Multiple myeloma” remains in the outer layer, but its link with “antineoplastic agents” appears. “Tunica intima,” “calcium channel agonists,” “immunosuppressive agents” exist in the outer layer of the network (Figure 3C and Table 1).

In the fourth stage (2000–2009), “vitamin D” is located at the very core of the network, with the largest node area and closer links to the surrounding nodes. “Cardiovascular diseases” and “vitamin d deficiency” are located in the second layer of the network diagram. “Vitamin D,” “vitamin D deficiency,” and “cardiovascular diseases” are linked by thicker lines. “Heart failure” is located further out in the network, but has a thicker line and closer relationship with “vitamin D.” “Diabetes mellitus, type 2,” “peripheral vascular diseases,” “hypertrophy, left ventricular,” “neoplasms,” “stroke,” “receptors, calcitriol” start to enter the outermost layer of the network. “Myocardial infarction,” which disappeared from the network in stages 2 and 3, reappears in this layer (Figure 3D and Table 1).

Stage 5 (2010–2020), in which the “vitamins” are located at the very heart of the network and have the largest node area, “Receptors, calcitriol” moves from the previous outer layer into the second layer and became one of the core subject terms here, with a larger node area. Core subject terms in this layer also include “cardiovascular diseases,” “vitamin D deficiency,” and “atherosclerosis.” The line between “cardiovascular diseases” and “vitamin D deficiency” is thicker. “Brain ischemia,” “renal insufficiency (chronic),” “metabolic syndrome” appear in the outer layer of the network (Figure 3E and Table 1).

#### 3.2.2. Vitamin D AND cardiovascular disease (0–18 years)

**FIGURE 4:**
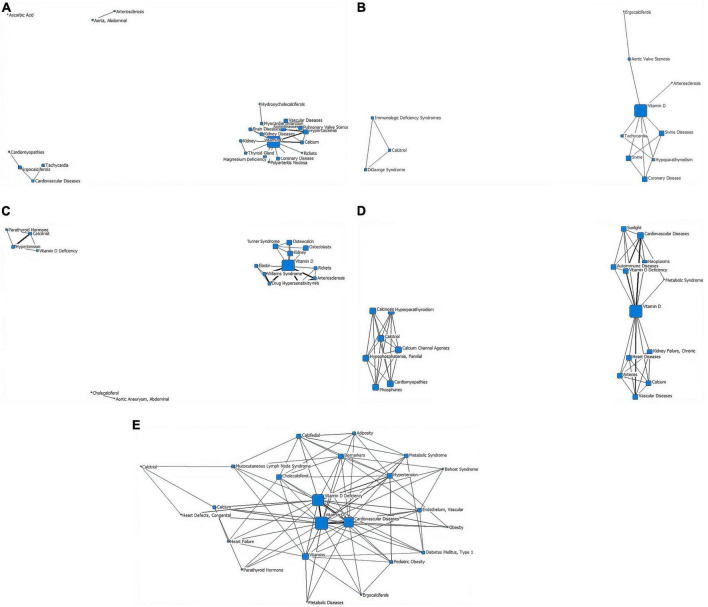
Social network of core subject terms of “vitamin D AND cardiovascular disease (0-18y)” in the PubMed database before December 2020. **(A)** 1979. **(B)** 1980-1989. **(C)** 1990-1999. **(D)** 2000-2009. **(E)** 2010-2020.

In the first stage (∼1979), the distribution of subject terms is more dispersed, converging into three categories and one isolated point. “Ascorbic acid” is the isolated point. “Arteriosclerosis” and “aorta, abdominal” are one category. “Ergocalciferols,” “cardiomyopathies,” “cardiovascular diseases,” “tachycardia” are as a group. The other subject headings are centered on the term “vitamin D.” The lines between “vitamin D” and “calcium” and “hypercalcemia” are the thickest (Figure 4A and Table 2).

In the second stage (1980–1989), research in this period converges into two categories. The first category is composed of the terms “calcitriol,” “immunologic deficiency syndromes,” and “DiGeorge syndrome,” with a triangular distribution in the network diagram. The second group, centered on “vitamin D,” has the largest nodal area and is associated upwards with “aortic valve Stenosis” and “arteriosclerosis” and downwards with “tachycardia” and “hypoparathyroidism”. The node area for “coronary disease” in this network is also relatively large. No cross-linking between the upper and lower branches of the second layer of the network (Figure 4B and Table 2).

In the third stage (1990–1999), research in this period converges into three main categories. The first group consists of “cholecalciferol” and “aortic aneurysm, abdominal”. The second group consists of “calcitriol,” “hypertension,” “vitamin D deficiency,” and “parathyroid hormone.” In the third category, “vitamin D” is the core term, the largest node area and the hub of the other terms, with a thicker line between “calcinosis” and “arteriosclerosis”. The nodes for “kidney,” “osteoblasts,” “osteocalcin,” “Turner syndrome,” and “Williams syndrome” (a developmental anomaly caused by the deletion of the proximal end of the long arm (7q11.23) of chromosome 7, which is mainly manifested by cardiovascular abnormalities, growth retardation, behavioral and psychological abnormalities, endocrine abnormalities, etc.) are also relatively large in the third category of the network (Figure 4C and Table 2).

In the fourth stage (2000–2009), research converges into two categories. The first group is centered on “calcitriol” and is associated with “calcium channel agonists,” “hypophosphatemia, familial,” and “calcinosis,” each with a similar node size. The second category is centered on “vitamin D” (it has a node area significantly larger than the other nodes) and with the thickest lines between “vitamin D deficiency,” “cardiovascular diseases,” and “vascular diseases.” The second type of network has no direct connection between the upper and lower parts of the subject terms. “Autoimmune diseases,” “sunlight,” “metabolic syndrome” are in the outer layer of the web (Figure 4D and Table 2).

In the fifth stage (2010–2020), the volume of literature increases and is clustered into a type of network diagram, centered on “vitamin D,” which has the largest node area and a triangular association with the second tier of subject terms “cardiovascular diseases” and “vitamin D deficiency.” “Pediatric obesity,” “diabetes mellitus (Type 1),” “endothelium, Vascular,” and “mucocutaneous lymph node syndrome” start to appear in the outer layer (Figure 4E and Table 2).

## 4. Discussion

With the help of bibliometric methods, we analyzed the evolving trends in the research on the association between vitamin D and cardiovascular disease and summarized the studies for the whole age group and for childhood respectively.

In terms of the growth of the literature, the results of the study with an all-age population showed that the literature in this field grew slowly before 2010 (157, 54, 84, and 211 publications that met the screening criteria with 336, 527, 343, and 879 main subject terms in stages 1–4, respectively), but after 2010 the research activity increased significantly, with 1,423 publications that met the screening criteria and 5,822 main subject terms from 2010 to 2020. The number of publications that met the screening criteria from 2010 to 2020 was 1,423, with 5,822 main subject terms, which was a good overall development. The results of the bibliometric analysis on children showed that the overall trend of research on the association between vitamin D and cardiovascular disease in children was similar to that of all-age studies, i.e., it entered an accelerated phase after 2010 (122 papers from 2010 to 2020, compared with 43 papers before 2009), but there were far fewer studies in children than in the all-age population over the same period (122/1423 in the last decade), and the acceleration time lagged behind that of all-age studies (Figure 1), suggesting that adult studies in this area are more mature and the results can be used as a reference for children’s studies.

Social network analysis of core subject terms showed that early all-age studies (stages 1–3) focused on the mechanisms of classical calcium and phosphorus metabolism regulated by vitamin D and cardiovascular diseases associated with these mechanisms, such as atherosclerosis and hypertension (although cardiovascular complications of pregnancy, growth problems in children and multiple myeloma have also been addressed). In stages 4–5, the research focus gradually extended to the exploration of the mechanisms of vitamin D in co-morbidities of cardiovascular disease (e.g., tumors, stroke, cerebral ischemia, and diabetes). The literature on the association between vitamin D and cardiovascular disease in children at stages 1–4 was few in number, with a lack of linkage between research hotspots and a multipolar distribution. In the last decade, there were a marked increase in the number of studies in this field and a strengthening of the links between research hotspots, with a growing interest in cardiovascular co-morbidities and a broadening of the scope, such as rickets, kidney disease, childhood obesity, diabetes and Kawasaki disease (also known as Mucocutaneous lymph node syndrome, is a childhood disease with systemic vasculitis as the main lesion, may lead to coronary artery dilation and coronary aneurysms). The overall trend was positive.

Vitamin D has been shown to be involved in blood pressure regulation, smooth muscle cell and endothelial cell physiological processes (8–11). Both the serum 25-(OH)D concentration and the incidence of cardiovascular disease exhibit geographic and seasonal fluctuations, suggesting a possible association between vitamin D and cardiovascular disease (12–16). Coronary artery disease (CAD), heart failure (HF), and atrial fibrillation (AF) are the three most common cardiovascular diseases and studies have confirmed that vitamin D deficiency is a very common co-morbidity of these three diseases (17, 18). Numerous studies have shown that vitamin D is associated with cardiovascular disease and its common co-morbidities such as nephropathy and metabolic syndrome (2–4, 12, 19–33). Vitamin D supplementation is no longer limited to the prevention and treatment of rickets, but has also been tried for the treatment of such diseases. Effective UV exposure, vitamin D fortification and food fortification are important means of preventing vitamin D deficiency (34–42). However, findings on the role of vitamin D in the treatment of cardiovascular disease and its co-morbidities appear to be inconsistent. Several teams have reported an inverse J or U curve effect between serum 25-(OH)D levels and cardiovascular disease risk and mortality, and have hypothesized that the adverse effect is associated with hypercalcemia (43–57). Some studies have also found no significant association between vitamin D concentrations and markers of subclinical atherosclerosis (48). The threshold at which serum 25-(OH)D increases cardiovascular mortality varies widely across studies (12.5–100 nmol/L) (49, 57). It is worth noting that in many previous study designs, limited by sample size and observation time, it was generally considered that dose-response analyses for vitamins were graded into 3–5 levels and then graded for disease association assessment, with cases of too low or too high vitamin D usually being considered extreme values (not included in the analysis). However, since changes in disease risk or treatment response are continuous rather than jumping, this design may lead to incomplete analytical conclusions. This may partly explain the conflicting results of previous studies on vitamin D and disease. Serum 25-(OH)D concentrations and blood pressure measurements were performed in 1074 Peruvian adolescents aged 13–15 years and found an association between uprightness intolerance and vitamin D deficiency in children (58). Children with vitamin D deficiency had elevated diastolic blood pressure and the degree of deficiency was positively correlated with blood pressure; Serum 25-(OH)D concentration showed a U-shaped relationship with systolic blood pressure, and an inverse J-shaped relationship with diastolic blood pressure and mean arterial pressure (58). It is speculated that vitamin D deficiency in childhood is associated with hypertension in adulthood. Conclusions for a U-shaped or inverse J-shaped effect of vitamin D have also been found in other studies of extraskeletal effects of vitamin D (59). A clear definition of nutritional criteria for vitamin D is the cornerstone of clinical intervention and scientific research. Research evidence suggests that for optimal skeletal effects of vitamin D, adult serum 25-(OH)D concentrations should be >50 nmol/L. From an overall health perspective, 25-(OH)D levels ≥75 nmol/L are considered ideal by the American Geriatrics Society, the Endocrine Society, the National Osteoporosis Foundation and the International Osteoporosis Foundation (60). Most national, regional and international scientific societies have published standards for vitamin D nutritional status and guidelines for prevention and treatment, but diagnostic and treatment recommendations for adolescents are often formulated with reference to data from adults or infants (61). In recent years, researchers have also begun to pay attention to and explore the optimal vitamin D threshold for achieving different extraskeletal effects (62–66). Studies have demonstrated that vitamin D supplementation in well-nourished individuals does not provide significant cardiovascular benefits (6). Therefore, the use of vitamin D in the management of cardiovascular disease requires a ‘cautious’ approach and individualization of doses. The design of studies relating vitamin D to the development and regression of cardiovascular disease should follow the principle of non-linear curves of biological responses to nutrients, i.e., as intake increases to what is generally considered to be the appropriate range, the risk decreases; beyond this range, pharmacological or toxic effects begin to emerge and the risk rises again, showing a U- or J-shaped dose-risk relationship. the left half of the U-shaped curve provides a clearer picture of the nutrient’s The left half of the U-curve gives a clearer picture of the health benefits of nutrients, with the benefits rising in an s-shaped curve as intake increases, but the dose-effect curve flattens out when intake is too small or too large. Nutrient studies often assume that a change in nutrient status will lead to a certain outcome, and as vitamin D absorption, conversion and biological effects vary between individuals studied, it is important to focus on the changes in nutritional status experienced by the study participants to reduce bias in the results of the study (51). We did not discuss the relationship between vitamin D level and arterial stiffness, which is associated with cardiovascular risk, morbidity, and mortality, this is the limitation of the study (67).

Based on the results of this study, research into the association between vitamin D and cardiovascular disease, particularly the mechanisms of association, and the identification of the role of vitamin D in common co-morbidities of cardiovascular disease and its value as a therapeutic tool, will be a focus of future research. Standardized vitamin D testing methods, long-term follow-up of large samples, and intervention strategies will provide a more reliable evidence-based basis for the prevention and treatment of vitamin D deficiency and related diseases.

## Data availability statement

The original contributions presented in this study are included in the article/supplementary material, further inquiries can be directed to the corresponding author.

## Author contributions

XL: preparing the tables and figures and drafting the manuscript. CWe: drafting the manuscript, reviewing and editing, conceptualization, and revising. FW and CWa: reviewing and editing the manuscript. All authors contributed to the article and approved the submitted version.
